# Body mass index modifies cardiovascular risk across blood pressure categories, particularly among underweight adults with hypertension: a 6.3 million‑person nationwide Japanese cohort study

**DOI:** 10.1038/s41440-026-02781-8

**Published:** 2026-08-31

**Authors:** Takahisa Kawano, Tomohisa Seki, Hiroaki Araie, Akiko Matsunaga, Naohiro Konoshita, Osamu Yamamura, Masamichi Ikawa

**Affiliations:** 1 Department of Emergency Medicine, University of Fukui Hospital, Fukui, Japan; 2 Department of Healthcare Information Management, The University of Tokyo Hospital, Tokyo, Japan; 3 Department of Hematology and Oncology, Faculty of Medical Sciences, University of Fukui, Fukui, Japan; 4 Department of Community Health Science, Faculty of Medical Sciences, University of Fukui, Fukui, Japan; 5 Department of Community Medicine, Faculty of Medical Sciences, University of Fukui, Fukui, Japan

**Keywords:** Body Mass Index, Digital Hypertension., Implemental Hypertension, Morning Hypertension

## Abstract

Co-occurring obesity and hypertension is associated with high cardiovascular risk, whereas the prognostic significance of underweight in the presence of hypertension remains uncertain. We examined whether the association between body mass index category and 10-year risk of major adverse cardiovascular events differed across blood pressure categories. Using the Japan Medical Claims Database from 2005 to 2023, we analyzed 6,262,298 adults without prior cardiovascular disease, chronic renal failure, dialysis, or antihypertensive medication use at baseline. Blood pressure was classified as normal (<120 and <80 mmHg), high-normal (120–129 and <80 mmHg), elevated (130–139 and/or 80–89 mmHg), stage 1 hypertension (140–159 and/or 90–99 mmHg), or stage 2/3 hypertension (≥160 and/or ≥100 mmHg). Body mass index was classified as underweight (<18.5 kg/m²), normal weight (18.5–24.9), overweight (25.0–29.9), or obesity (≥30.0). The primary outcome was major adverse cardiovascular events within 10 years. Multivariable Cox proportional hazards models estimated hazard ratios for combined blood pressure and body mass index categories, using normal blood pressure with normal weight as the reference. During a median follow-up of 1,183 days, 34,034 participants experienced major adverse cardiovascular events. Underweight showed a marked blood pressure-dependent increase in risk, with the hazard ratio rising from 1.14 (95% confidence interval, 1.07–1.22) in normal blood pressure to 5.01 (4.23–5.93) in stage 2/3 hypertension. The interaction between blood pressure category and body mass index category was significant. These findings suggest that higher blood pressure may amplify the adverse prognostic significance of underweight.

**Figure:**
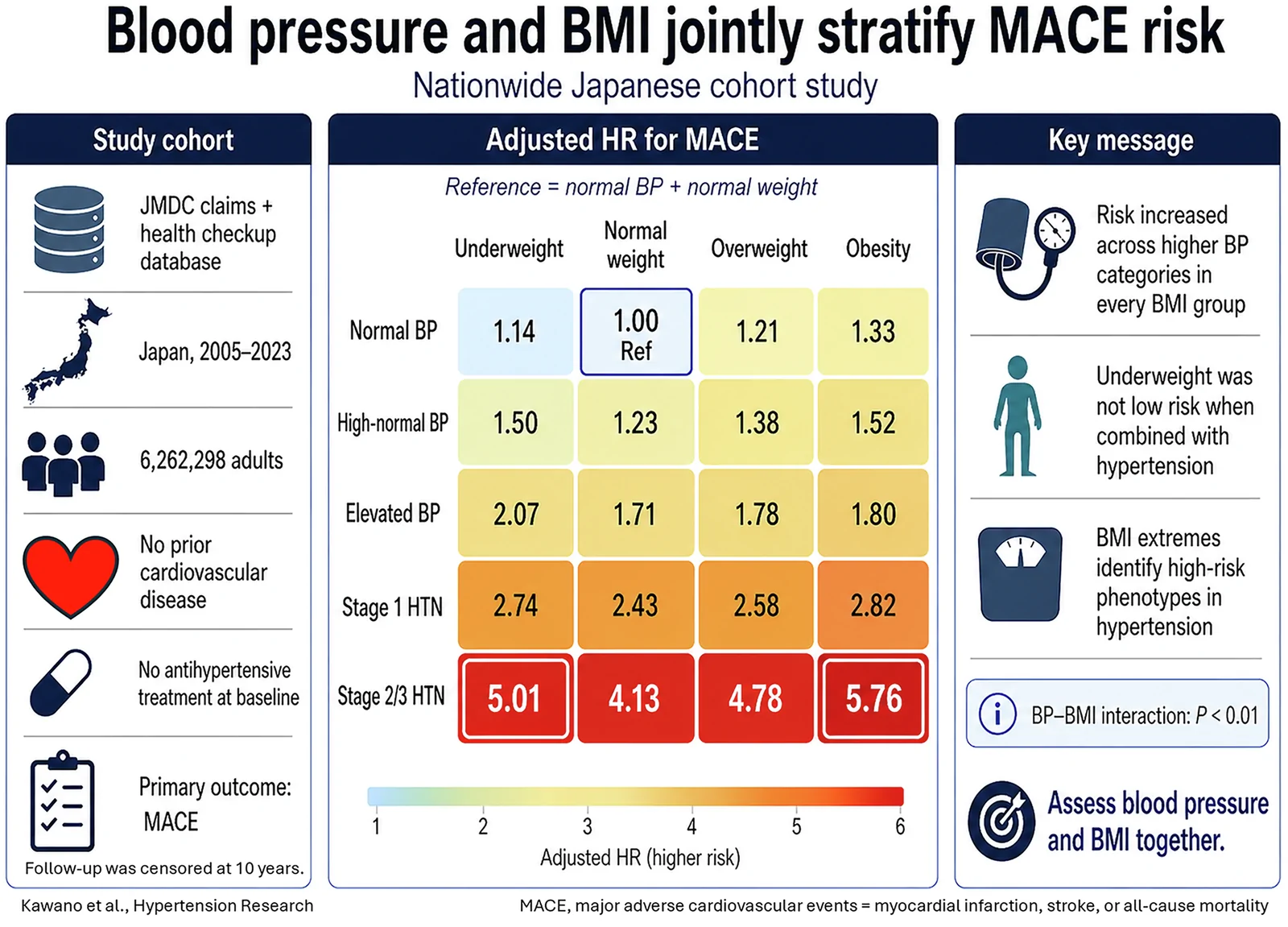

## Introduction

Hypertension is a major global health concern, affecting nearly one-third of adults—approximately 1.3 billion people as of 2019 [1]. Elevated blood pressure (BP) is the leading modifiable risk factor for cardiovascular morbidity and mortality, accounting for an estimated 10.8 million deaths annually or nearly 19% of all global deaths [2]. Furthermore, hypertension accounts for approximately 40% of all ischemic heart disease-related deaths and nearly 50% of stroke-related deaths [1, 3].

Obesity coexists with hypertension, and the prevalence of both conditions has increased simultaneously across populations worldwide [4]. Observational studies have consistently demonstrated that the coexistence of hypertension and obesity substantially increases cardiovascular risk beyond that conferred by either factor independently [5]. Recent evidence suggests an increased cardiovascular risk among individuals classified as underweight [6]; however, these individuals have generally been excluded from studies that have primarily focused on populations with obesity [7]. As a result, it remains unclear whether the association between the body mass index (BMI) and cardiovascular risk is consistent across different levels of blood pressure, or whether BMI modifies the effect of hypertension on cardiovascular outcomes.

We hypothesized that the association between BMI category and 10-year risk of major adverse cardiovascular events (MACEs) differs across blood pressure (BP) categories. Therefore, using a nationwide health claims database, we evaluated MACE risk across combined BP–BMI categories defined according to BMI status and BP categories based on the Japanese Society of Hypertension Guidelines for the Management of Hypertension [8]. We further tested whether the association between BMI category and MACE risk varied by BP category, thereby assessing the joint relevance of BP and BMI for cardiovascular risk stratification.

Point of view

- **Clinical relevance**
  Underweight was associated with relatively low crude MACE incidence when BP was normal, but MACE risk rose markedly with elevated BP or hypertension, supporting joint assessment of BP and BMI rather than an obesity-centered approach.
- **Future direction**
  Future studies should determine why underweight patients with hypertension have higher MACE risk by characterizing their clinical backgrounds, including unintentional weight loss, nutritional status, body composition, frailty, comorbidities, and cause-specific outcomes.
- **Consideration for the Asian population**
  In Asian health-check settings, preventive attention should extend beyond obesity with high BP to underweight with elevated BP or hypertension, prompting additional assessment and appropriate intervention.

## Methods

### Dataset description

This retrospective observational study was conducted using data from the JMDC Claims Database from January 2005 to October 2023 (*n* = 8,783,784) [9] The database contains health insurance claims data from more than 60 insurers and provides comprehensive information on workplace employees, and includes annual health checkup records with demographic details, medical history, medication use, hospital claims coded according to the International Classification of Diseases 10th Revision (ICD-10), and mortality data.

### Study participants

The participants were adults aged 18–65 years. For the selection thereof, we excluded records with missing data on BP, antihypertensive medication use, or BMI. Additionally, individuals with a history of myocardial infarction (MI), stroke, chronic renal failure, dialysis use, or those taking antihypertensive medications at baseline were excluded from the analysis.

### Measurements

BP was measured by healthcare professionals using a standard sphygmomanometer or an automated device. Although Japanese guidelines recommend measuring BP twice consecutively in a seated position, actual measurement protocols vary depending on the responsible health check-up organizations [10]. As indicated in recent reviews of Japanese real-world data, the BP data may include values from a single measurement, or the lower value among multiple measurements [10, 11]. Additional data, including BMI, waist circumference measured at the umbilicus, and medical history assessed via self-administered questionnaires, were obtained using standardized protocols [11]. Fasting laboratory values, defined as samples collected 10 hours after the last meal, included lipid profiles and glycemic indices. Smoking status (current/non-smoker) and alcohol consumption (daily/less than daily) were self-reported by the participants. High waist circumference was defined as ≥85 and ≥90 cm for males and females, respectively [12]. Diabetes was defined as having a fasting glucose level of ≥126 mg/dL or the use of glucose-lowering medications. Dyslipidemia was defined as having low-density lipoprotein cholesterol (LDL), high-density lipoprotein cholesterol (HDL), and triglyceride levels of ≥140 mg/dL, <40 mg/dL, and ≥150 mg/dL, respectively, or the use of lipid-lowering medications.

### Outcomes

Clinical outcomes were identified from the hospital claims data collected between January 2005 and October 2023. The primary outcome was a composite of major adverse cardiovascular events (MACEs) defined as the first occurrence of MI, stroke defined as a composite of ischemic stroke and intracerebral hemorrhage (ICH), or all-cause mortality [13]. Because relying solely on ICD diagnostic codes from claims data is insufficient for defining cardiovascular diseases, we used validated algorithms combining diagnosis, prescription, and procedure codes. When Diagnosis Procedure Combination (DPC) codes were available, the diagnoses were made based on DPC codes; the positive predictive value (PPV) for MI in DPC claims was 78.7 to 93.8% [14, 15] and for ischemic stroke or ICH in DPC claims was 85.7% to 89.9% [14, 16]. In the absence of a DPC code, we used a combination of ICD-10 codes and other claims data. Specifically, MI was identified by the presence of an ICD-10 code (I21 or I22) combined with codes for hospitalization and antiplatelet drugs [17]. Ischemic stroke was identified using an ICD diagnosis code (I63) combined with prescription drugs (antiplatelet drugs, anticoagulants, heparin, anti-thrombin, aspirin, cerebral metabolism activator, or thrombolytic agents), claim codes for ultrasonography, and rehabilitation [17]. ICH was identified using an ICD diagnosis code (I60, I61, or I62) along with claim codes for MRI or CT imaging and rehabilitation [17]. The PPVs for each diagnosis were 87.0% (78.3–93.1) for MI, 44.4% (32.7–56.6%) for ischemic stroke, and 46.1% (34.5–57.9%) for ICH [17]. Furthermore, all-cause mortality was defined as death if the specified reason for the loss of health insurance eligibility was death [18–20]. Outcomes were recorded for up to 10 years after the baseline health checkup, and events occurring beyond this period were censored at 10 years.

### Statistical analysis

Descriptive statistics are presented as means with standard deviations, medians with interquartile ranges (IQRs) for skewed distributions, and proportions where applicable. Statistical significance was set at *P* < 0.05. All statistical analyses were performed using the R software version 4.4.0 (R Foundation for Statistical Computing, Vienna, Austria).

The participants were first categorized by baseline BP values according to the Japanese Society of Hypertension Guidelines 2025 [8]. These categories included normal BP, defined as untreated systolic BP and diastolic BP of <120 and <80 mmHg, respectively; high-normal BP, 120–129 and <80 mmHg, respectively; elevated BP, 130–139 and/or 80–89 mmHg, respectively; stage 1 hypertension, 140–159 and/or 90–99 mmHg, respectively and stage 2/3 hypertension, ≥160 and/or ≥100 mmHg, respectively. Within each BP category, participants were further subdivided based on BMI, according to the World Health Organization’s classifications as follows: underweight (<18.5 kg/m^2^), normal weight (18.5–24.9 kg/m^2^), overweight (25.0–29.9 kg/m^2^), and obese (≥30 kg/m^2^) [21].

Cox proportional hazards regression models were used to evaluate the association between the combined BP–BMI categories and the subsequent risk of MACEs. Hazard ratios (HRs) with corresponding 95% confidence intervals (CIs) were estimated using models constructed from multiple imputed datasets, compared with normal BP and normal weight categories as a reference group. The models were adjusted for predefined confounding variables, including age, sex, waist circumference, diabetes, dyslipidemia, cigarette smoking status, and alcohol consumption status, based on the established clinical relevance and associations with BP and cardiovascular outcomes [19]. Missing covariate data were assumed to be missing at random and imputed via multivariate imputation using the chained equations method, employing 20 imputations, as recommended by Aloisio et al. [20]. The combined HRs and standard errors across the imputed datasets were pooled using Rubin’s rules [22]. To examine whether the association between blood pressure category and subsequent risk of MACEs differed according to BMI category, we additionally fitted multivariable Cox proportional hazards models using the same outcome and covariate adjustment as in the primary analysis, with blood pressure category, BMI category, and their interaction term included in the model. The significance of the interaction was assessed by comparing models with and without the interaction term.

Several sensitivity analyses were conducted. First, sex-stratified analyses were performed to evaluate whether the association between BP–BMI categories and the risk of MACEs differed between men and women. In addition, age-stratified analyses were conducted by categorizing participants into three groups (<40, 40–49, and ≥50 years) to assess potential effect modification by age. In each stratified analysis, Cox proportional hazards models were fitted using the same covariate adjustment set as in the primary analysis. Second, A lag analysis was performed as a sensitivity analysis to reduce potential reverse causation. Specifically, participants who remained event-free and were still under follow-up at 3 years were included in this analysis. Cox proportional hazards models were then applied using the same covariate adjustment as in the primary analysis. Finally, we separately evaluated the associations of the BP-BMI categories with each outcome (MI, stroke, and all-cause mortality) using adjusted models based on multiple imputed data.

### Ethical approval

This study adhered to the principles outlined in the Declaration of Helsinki, was conducted in accordance with the ethical guidelines of our institution, and was approved by the Ethical Committee of the University of Fukui (Approval number: 20240157). The requirement for obtaining informed consent was waived as all data used from the JMDC Claims Database were anonymized and de-identified.

## Results

**Fig. 1:**
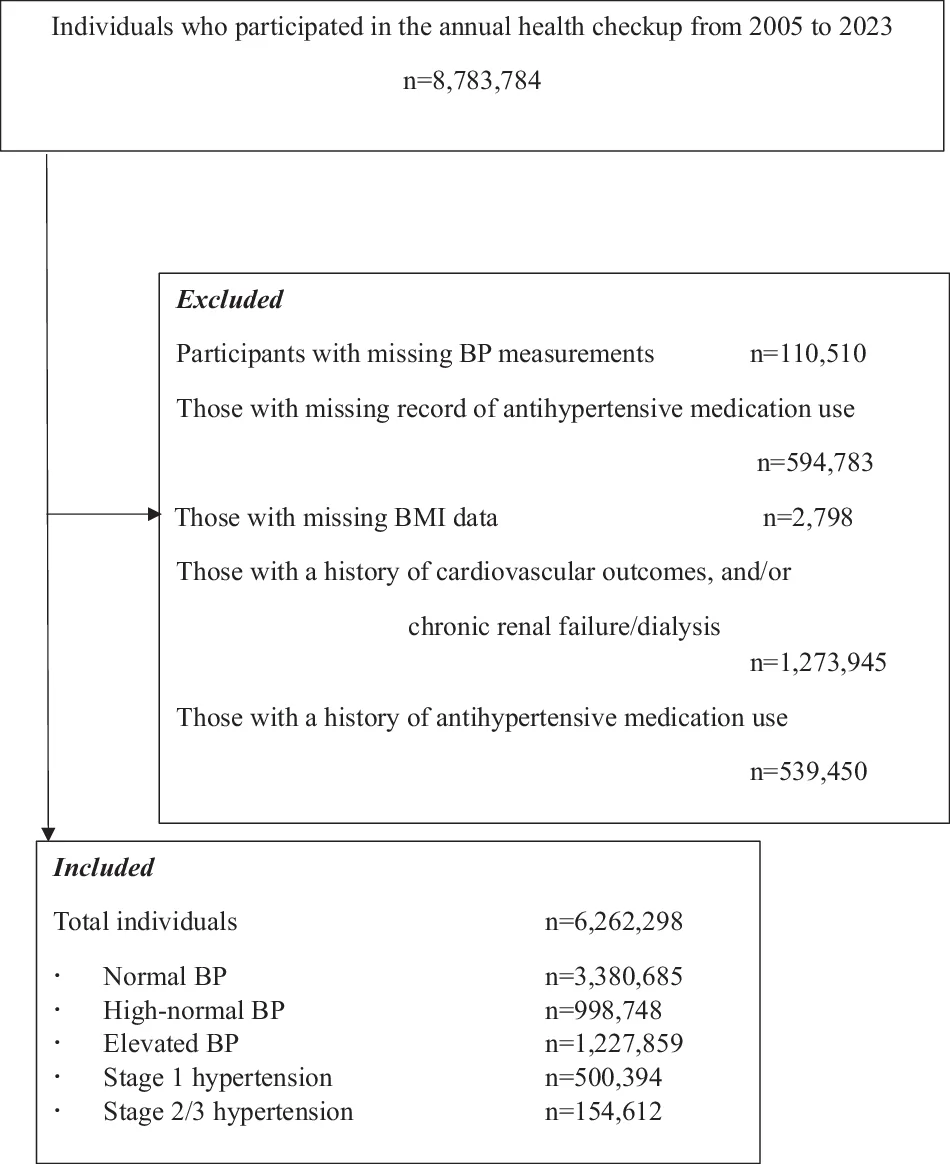
Study flow chart

Of the initial 8,773,784 participants identified, 110,510, 594,783 and 2798 were excluded owing to missing BP measurements, records of anti-hypertensive medication use and BMI data, respectively. We then excluded 1,273,945 participants with a documented history of cardiovascular outcomes or chronic renal failure/dialysis at baseline and 239,450 who were receiving antihypertensive medications. The final sample comprised 6,262,298 participants (Fig. 1).

**Fig. 2:**
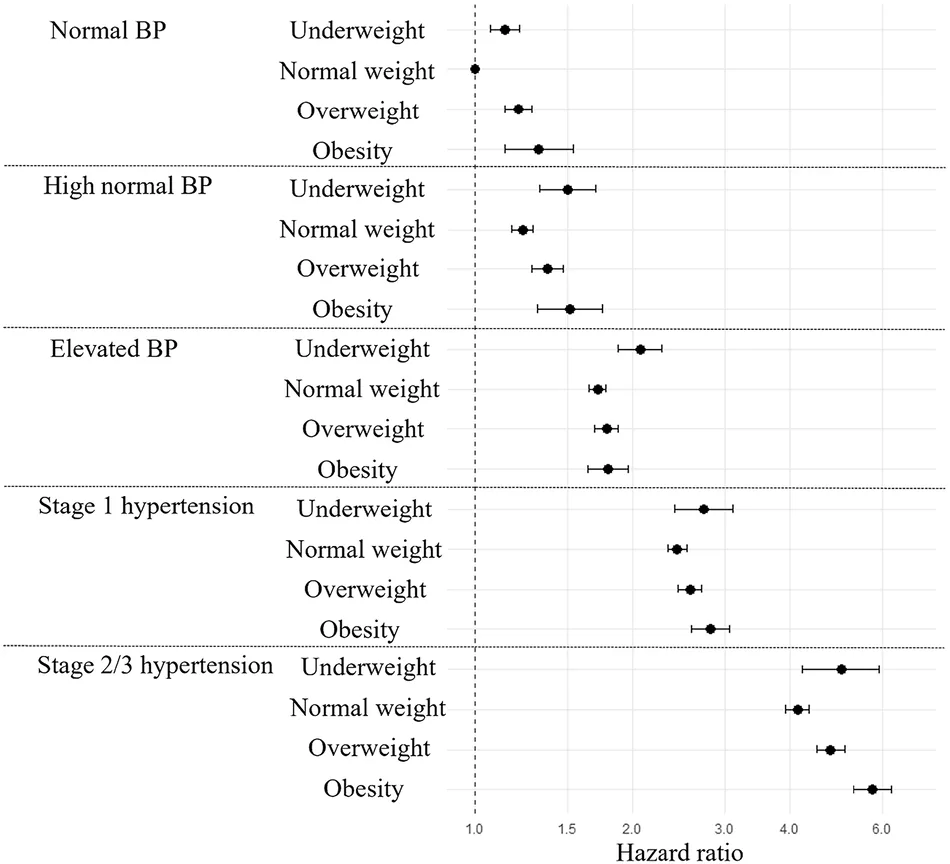
Forest plot of the adjusted hazard ratio for MACEs. Participants were categorized as having normal BP (untreated systolic and diastolic BPs of <120 and <80 mmHg), high normal BP (120–129 and <80 mmHg), elevated BP (130–139 and 80–89 mmHg), stage 1 hypertension (140–159 and 90–99 mmHg), or stage 2/3 hypertension (≥160 and/or ≥100 mmHg), and were further subdivided based on BMI as follows: underweight (<18.5 kg/m²), normal weight (18.5–24.9 kg/m²), overweight (25.0–29.9 kg/m²), and obesity (≥30 kg/m²). Points indicate adjusted hazard ratios, and horizontal lines indicate 95% confidence intervals. MACEs, major adverse cardiovascular events

Participants were categorized into four groups according to baseline BP measurements as follows: normal BP (*n* = 3,380,685), high-normal BP (998,748), elevated BP (*n* = 1,227,859), stage 1 hypertension (*n* = 500,394), and stage 2/3 hypertension (*n* = 154,612). All participants in the high-normal BP, elevated BP, stage 1 hypertension, and stage 2/3 hypertension categories were predominantly male and generally older than those in the normal BP category. These groups demonstrated a progressively greater proportion classified as having overweight or obesity, as well as a higher prevalence of increased waist circumference than the normal BP group. (Fig. 2)

Participants in the high-normal, elevated, stage 1, and stage 2/3 hypertension categories also had higher baseline values of fasting glucose, glycated hemoglobin, LDL cholesterol, and triglycerides, and lower HDL cholesterol levels than those of participants in the normal BP group. Furthermore, the prevalence of diabetes, dyslipidemia, cigarette smoking, and alcohol consumption progressively increased across the BP categories from normal BP to stage 2/3 hypertension (Table 1)

**Table 1:** Patient demographics

		Normal BP	Elevated BP	High-normal BP	Stage-1-hypertension	Stage2/3-hypertension
***Characteristic***	missing	(*n* = 3,380,685)	(*n* = 1,227,859)	(*n* = 998,748)	(*n* = 500,394)	(*n* = 154,612)
Male sex, *n* (%)	0	1,473,123 (43.6)	834,357 (68)	652,800 (65.4)	346,102 (69.2)	104,646 (67.7)
Age, years (IQR)	0	40 (30–47)	45 (37–53)	40 (29– 49)	49 (41–57)	50 (44–57)
***BMI category***	0					
Underweight	0	522,805 (15.5)	68,145 (5.5)	70,921 (7.1)	18,860 (3.8)	4,966 (3.2)
Normal weight	0	2,455,200 (72.6)	747,782 (60.9)	675,121 (67.6)	265,626 (53.1)	71,205 (46.1)
Overweight	0	357,427 (10.6)	324,729 (26.4)	208,485 (20.9)	156,202 (31.2)	52,432 (33.9)
Obese	0	45,253 (1.3)	87,203 (7.1)	44,221 (4.4)	59,706 (11.9)	26,009 (16.8)
High waist circumference, *n* (%)	496,954	409,941 (13.4)	436,095 (37.7)	251,058 (27.9)	227,432 (47.1)	81,827 (53.9)
Diabetes, *n* (%)	1,191,436	43,036 (1.6)	48,731 (4.9)	24,656 (3.3)	31,248 (7.7)	12,466 (9.9)
Dyslipidemia, *n* (%)	214,534	860,110 (26.5)	564,304 (47.1)	356,889 (37.4)	269,999 (54.8)	91,907 (60)
Cigarette smoking, *n* (%)	13,235	715,789 (21.2)	336,757 (27.5)	259,122 (26)	142,261 (28.6)	46,872 (30.4)
Alcohol consumption, *n* (%)	91,532	465,862 (14)	313,248 (26)	177,436 (18.1)	153,578 (31.3)	52,416 (34.4)
	0					
***Laboratory data***	0					
Glucose, mg/dL (IQR)	1,205,027	89 (84–94)	93 (87–100)	91 (85– 97)	96 (89–104)	98 (91–107)
HbA1c, % (IQR)	862,906	5.3 (5.1–5.5)	5.4 (5.2–5.7)	5.4 (5.2– 5.6)	5.5 (5.3–5.8)	5.5 (5.3–5.8)
LDL, mg/dL (IQR)	212,533	111 (92–132)	123 (102–145)	117 (97–139)	127 (106–150)	131 (109–154)
HDL, mg/dL (IQR)	208,411	65 (55–76)	60 (50–72)	61 (51–72)	59 (49–72)	59 (49–72)
Triglycerides, mg/dL (IQR)	205,536	68 (49–98)	94 (65–141)	82 (57–122)	105 (72–158)	114 (77–173)

Participants were categorized as having normal BP (untreated systolic and diastolic BPs of <120 and <80 mmHg), high normal BP (120–129 and <80 mmHg), elevated BP (130–139 and 80–89 mmHg), stage 1 hypertension (140–159 and 90–99 mmHg), or stage 2/3 hypertension (≥160 and/or ≥100 mmHg), and were further subdivided based on BMI as follows: underweight (<18.5 kg/m²), normal weight (18.5–24.9 kg/m²), overweight (25.0–29.9 kg/m²), and obesity (≥30 kg/m²)

*IQR* interquartile range, *HbA1c* glycated hemoglobin, *LDL* low-density lipoprotein cholesterol, *HDL* high-density lipoprotein cholesterol, *BP* blood pressure, *BMI* body mass index

### Incidence of MACEs according to BP and BMI categories

During a median follow-up of 1183 (IQR: 479–2086) days, 34,034 MACEs occurred among 6,262,298 participants. The incidence rate of MACEs increased progressively across BP categories in all BMI groups. Among participants with underweight, the incidence rate rose from 0.21 per 1000 person-years in the normal BP category to 2.84 per 1000 person-years in the stage 2/3 hypertension category. Within the normal BP category, participants with underweight had the lowest incidence rate of MACEs. In contrast, in the stage 2/3 hypertension category, participants with underweight had a higher incidence rate than those with normal weight and obesity: 2.84 versus 2.69 and 2.69 per 1000 person-years, respectively (Table 2).

**Table 2:** Frequency of events and incidence rate for MACEs

	Number	Events (%)	Incidence rate
***Normal BP***			
Underweight	522,805	1071 (0.2)	0.21
Normal weight	2,455,200	6357 (0.3)	0.26
Overweight	357,427	1542 (0.4)	0.43
Obese	45,253	181 (0.4)	0.4
***Elevated BP***			
Underweight	68,145	448 (0.7)	0.66
Normal weight	747,782	5610 (0.8)	0.75
Overweight	324,729	2770 (0.9)	0.86
Obese	87,203	577 (0.7)	0.66
***High-normal BP***			
Underweight	70,921	262 (0.4)	0.37
Normal weight	675,121	2718 (0.4)	0.4
Overweight	208,485	1087 (0.5)	0.52
Obese	44,221	206 (0.5)	0.47
***Stage 1 hypertension***			
Underweight	18,860	251 (1.3)	1.34
Normal weight	265,626	3714 (1.4)	1.41
Overweight	156,202	2290 (1.5)	1.48
Obese	59,706	701 (1.2)	1.18
***Stage 2/3 hypertension***			
Underweight	4966	138 (2.8)	2.84
Normal weight	71,205	1882 (2.6)	2.69
Overweight	52,432	1541 (2.9)	3
Obese	26,009	688 (2.6)	2.69

The incidence rate was per 1000 person-years. Participants were categorized as having normal BP (untreated systolic and diastolic BPs of <120 and <80 mmHg), high normal BP (120–129 and <80 mmHg), elevated BP (130–139 and 80–89 mmHg), stage 1 hypertension (140–159 and 90–99 mmHg), or stage 2/3 hypertension (≥160 and/or ≥100 mmHg), and were further subdivided based on BMI as follows: underweight (<18.5 kg/m²), normal weight (18.5–24.9 kg/m²), overweight (25.0–29.9 kg/m²), and obesity (≥30 kg/m²)

*MACEs* major adverse cardiovascular events, *BMI* body mass index, *BP* blood pressure

### Cox proportional hazards models

In the adjusted Cox proportional hazards models, the risk of MACEs increased progressively across higher BP categories within each BMI group (Table 3). Compared with participants with normal BP and normal weight, participants with underweight showed an increasing risk gradient from normal BP to stage 2/3 hypertension: HR 1.14 (95% CI, 1.07–1.22) in normal BP, 1.50 (95% CI, 1.33–1.70) in high-normal BP, 2.07 (95% CI, 1.88–2.27) in elevated BP, 2.74 (95% CI, 2.41–3.11) in stage 1 hypertension, and 5.01 (95% CI, 4.23–5.93) in stage 2/3 hypertension.

**Table 3:** Adjusted HR for MACEs in the main, sex- or age–stratified analysis, and the lag analyses

	Main analysis	Sex-stratified analysis		Age-stratified analysis			Lag analysis
		Male	Female	<40	40-49	>=50	
BP-BMI category	Adjusted HR (95% CI)	Adjusted HR (95% CI)	Adjusted HR (95% CI)	Adjusted HR (95% CI)	Adjusted HR (95% CI)	Adjusted HR (95% CI)	Adjusted HR (95% CI)
***Normal BP***							
Underweight	1.14 (1.07–1.22)	1.15 (1.04–1.27)	1.12 (1.03–1.22)	1.10 (0.95–1.28)	0.95 (0.85–1.07)	1.03 (0.94–1.13)	1.07 (0.97–1.17)
Healthy weight	1.00 (Reference)	1.00 (Reference)	1.00 (Reference)	1.00 (Reference)	1.00 (Reference)	1.00 (Reference)	1.00 (Reference)
Overweight	1.21 (1.14–1.28)	1.21 (1.13–1.30)	1.21 (1.07–1.37)	0.97 (0.80–1.18)	1.20 (1.09–1.32)	1.12 (1.03–1.22)	1.19 (1.10–1.29)
Obesity	1.33 (1.14–1.54)	1.46 (1.23–1.73)	1.01 (0.73–1.40)	1.05 (0.69–1.60)	1.30 (1.05–1.61)	1.00 (0.78–1.28)	1.39 (1.14–1.70)
***High-normal BP***							
Underweight	1.50 (1.33–1.70)	1.57 (1.32–1.86)	1.52 (1.27–1.82)	1.90 (1.39–2.60)	1.43 (1.11 - 1.84)	1.34 (1.14–1.57)	1.20 (0.99–1.45)
Healthy weight	1.23 (1.18–1.29)	1.19 (1.12–1.25)	1.47 (1.35–1.60)	1.35 (1.18–1.53)	1.34 (1.24–1.46)	1.32 (1.24–1.40)	1.19 (1.12–1.27)
Overweight	1.38 (1.29–1.47)	1.40 (1.30–1.51)	1.34 (1.13–1.57)	1.47 (1.20–1.80)	1.42 (1.27–1.59)	1.29 (1.18–1.42)	1.42 (1.30–1.55)
Obesity	1.52 (1.32–1.75)	1.52 (1.29–1.79)	1.60 (1.20–2.15)	1.17 (0.79–1.74)	1.39 (1.12–1.73)	1.49 (1.21–1.84)	1.49 (1.24–1.81)
***Elevated BP***							
Underweight	2.07 (1.88–2.27)	2.24 (1.97–2.54)	1.97 (1.70–2.28)	2.06 (1.46–2.90)	2.11 (1.74–2.55)	1.78 (1.58–2.01)	2.14 (1.88–2.44)
Healthy weight	1.71 (1.65–1.78)	1.68 (1.61– 1.75)	1.93 (1.79–2.07)	1.85 (1.63–2.09)	2.02 (1.89–2.15)	1.70 (1.62–1.79)	1.75 (1.67–1.84)
Overweight	1.78 (1.69–1.87)	1.79 (1.69–1.89)	1.75 (1.54–1.98)	1.78 (1.48–2.13)	2.00 (1.85–2.18)	1.53 (1.43–1.64)	1.77 (1.65–1.90)
Obesity	1.80 (1.64–1.96)	1.85 (1.68–2.05)	1.64 (1.31– 2.04)	1.99 (1.55–2.56)	1.85 (1.62–2.11)	1.32 (1.14–1.52)	1.86 (1.65–2.09)
***Stage 1 hypertension***							
Underweight	2.74 (2.41 - 3.11)	2.73 (2.31–3.23)	2.97 (2.44–3.61)	3.93 (2.04–7.58)	3.80 (2.89–4.99)	2.29 (1.98–2.65)	2.11 (1.73–2.58)
Healthy weight	2.43 (2.33–2.54)	2.34 (2.23–2.45)	2.92 (2.68–3.19)	2.98 (2.45–3.62)	3.50 (3.25–3.78)	2.29 (2.17–2.42)	2.27 (2.14–2.40)
Overweight	2.58 (2.44–2.72)	2.57 (2.42–2.72)	2.65 (2.32–3.04)	2.93 (2.34–3.67)	3.19 (2.92–3.49)	2.10 (1.96–2.25)	2.36 (2.19 - 2.54)
Obesity	2.82 (2.60–3.06)	2.77 (2.52–3.03)	3.26 (2.70–3.95)	3.47 (2.71–4.43)	2.93 (2.59–3.33)	1.97 (1.73–2.23)	2.68 (2.39–3.00)
***Stage 2/3 hypertension***							
Underweight	5.01 (4.23–5.93)	4.79 (3.83–5.99)	5.71 (4.40–7.41)	8.06 (2.59–25.06)	8.21 (5.76–11.70)	4.04 (3.32–4.92)	3.65 (2.76–4.82)
Healthy weight	4.13 (3.92–4.36)	4.06 (3.83–4.31)	4.47 (3.99–5.02)	9.80 (7.55–12.72)	6.40 (5.80–7.06)	3.68 (3.45–3.92)	3.26 (3.01–3.53)
Overweight	4.78 (4.50–5.08)	4.73 (4.43–5.06)	5.17 (4.44–6.01)	9.43 (7.35–12.10)	6.27 (5.67–6.92)	3.47 (3.20–3.75)	3.86 (3.53–4.22)
Obesity	5.76 (5.30–6.26)	5.93 (5.41–6.50)	5.32 (4.32–6.54)	10.54 (8.30–13.39)	6.17 (5.46–6.98)	3.16 (2.76–3.63)	4.67 (4.13–5.28)

Participants were categorized as having normal BP (untreated systolic and diastolic BPs of <120 and <80 mmHg), high-normal BP (120–129 and <80 mmHg), elevated BP (130–139 and/or 80–89 mmHg), stage 1 hypertension (140–159 and/or 90–99 mmHg), or stage 2/3 hypertension (≥160 and/or ≥100 mmHg), and were further subdivided based on BMI as follows: underweight (<18.5 kg/m²), normal weight (18.5–24.9 kg/m²), overweight (25.0–29.9 kg/m²), and obesity (≥30 kg/m²). The main and lag models were adjusted for age, sex, waist circumference, diabetes, dyslipidemia, smoking status, and alcohol consumption. Sex-stratified models excluded sex from the adjustment set, and age-stratified models included age as a continuous covariate within each age stratum. The lag analysis included participants who remained event-free and were still under follow-up at 3 years. *MACEs* major adverse cardiovascular events, *HR* hazard ratio, *CI* confidence interval, *BMI* body mass index, *BP* blood pressure

Within BP categories, underweight was associated with higher adjusted HRs than normal weight in all BP strata. In the elevated BP group, underweight showed the highest adjusted HR among BMI categories: HR 2.07 (95% CI, 1.88–2.27), compared with 1.71 (95% CI, 1.65–1.78) for normal weight, 1.78 (95% CI, 1.69–1.87) for overweight, and 1.80 (95% CI, 1.64–1.96) for obesity. In the stage 1 hypertension group, the adjusted HR for underweight was 2.74 (95% CI, 2.41–3.11), which was higher than that for normal weight and overweight. In the stage 2/3 hypertension group, underweight was associated with an elevated risk of MACEs, with an adjusted HR of 5.01 (95% CI, 4.23–5.93), exceeding the HRs for normal weight and overweight but not obesity, which showed the highest HR in this stratum: HR 5.76 (95% CI, 5.30–6.26). The interaction between blood pressure category and BMI category was tested by comparing multivariable Cox proportional hazards models with and without the interaction term. The overall interaction was statistically significant (P for interaction < 0.01).

### Sensitivity analyses

In sex-stratified analyses, underweight combined with higher BP categories was consistently associated with elevated MACE risk in both men and women (Table 3). Among men, the adjusted HR for underweight increased from 1.15 (95% CI, 1.04–1.27) in the normal BP group to 4.79 (95% CI, 3.83–5.99) in the stage 2/3 hypertension group. Among women, the corresponding HRs were 1.12 (95% CI, 1.03–1.22) and 5.71 (95% CI, 4.40–7.41), respectively. In women with stage 2/3 hypertension, underweight showed a higher HR than obesity: HR 5.71 (95% CI, 4.40–7.41) versus 5.32 (95% CI, 4.32–6.54). Age-stratified analyses also showed a consistent excess risk associated with underweight in hypertensive participants. In participants aged 40–49 years, underweight with stage 2/3 hypertension had the highest adjusted HR among BMI categories: HR 8.21 (95% CI, 5.76–11.70). A similar pattern was observed in participants aged ≥50 years, in whom underweight with stage 2/3 hypertension was associated with an adjusted HR of 4.04 (95% CI, 3.32–4.92), higher than the corresponding HRs for normal weight, overweight, and obesity. In participants aged <40 years, HRs were high across BMI categories in the stage 2/3 hypertension group, although underweight was not the highest-risk BMI category. In the lag analysis, including participants who remained event-free and were still under follow-up at 3 years, the overall pattern remained similar. Underweight continued to show elevated risk across BP categories, with HRs of 1.07 (95% CI, 0.97–1.17) in normal BP, 1.20 (95% CI, 0.99–1.45) in high-normal BP, 2.14 (95% CI, 1.88–2.44) in elevated BP, 2.11 (95% CI, 1.73–2.58) in stage 1 hypertension, and 3.65 (95% CI, 2.76–4.82) in stage 2/3 hypertension. The results for the individual outcomes are presented in the Supplementary Table 1.

## Discussion

In this large nationwide cohort study of 6,262,298 adults, we examined the joint association of BP and BMI categories with the risk of MACEs. The incidence rate of MACEs increased progressively across higher BP categories in all BMI groups. Although participants with underweight had the lowest crude incidence rate in the normal BP category, their risk increased markedly with advancing BP categories. In the multivariable-adjusted Cox models, underweight was associated with a higher risk of MACEs than normal weight within each BP stratum, and this excess risk became more apparent among participants with elevated BP or hypertension. Participants with underweight and stage 2/3 hypertension showed a substantially elevated risk of MACEs compared with those with normal BP and normal weight. The significant interaction between blood pressure category and BMI category supports the presence of effect modification by BMI, consistent with the finding that the excess cardiovascular risk associated with higher blood pressure was not uniform across BMI categories. The stratified analyses generally supported the main findings. In both men and women, underweight combined with higher BP categories was consistently associated with elevated MACE risk. Similar patterns were observed across age strata, with a particularly high risk among participants with underweight and stage 2/3 hypertension. In the lag analysis, the association between underweight, higher BP categories, and increased MACE risk was attenuated but remained evident. Overall, underweight status should not be interpreted as a low-risk phenotype when accompanied by elevated BP or hypertension, and the combination of underweight and severe hypertension may identify a clinically important high-risk subgroup.

Although underweight has previously been associated with increased cardiovascular events [6, 23, 24] and mortality [25, 26], our findings refine this evidence by showing that the cardiovascular risk associated with underweight may depend substantially on BP level among working-age adults. In the main analysis, participants with underweight did not show a high crude incidence of MACEs when BP was normal; however, their risk increased markedly with advancing BP categories. Thus, the key implication is not simply that low BMI and high BP are each harmful, but that the prognostic meaning of underweight may vary according to BP level. Underweight individuals with hypertension may therefore represent a distinct high-risk subgroup that could be overlooked if cardiovascular prevention strategies focus mainly on obesity or excess body weight.

Stratified analyses suggested that the excess risk associated with underweight and hypertension was not uniform across sex and age groups. Among men, obesity showed higher HRs than underweight in the stage 1 and stage 2/3 hypertension categories, which may be partly explained by the tendency of men to accumulate more visceral fat associated with increased cardiometabolic risk, and to exhibit more severe inflammation-driven vascular dysfunction and cardiac remodeling in response to obesity [27, 28]. In contrast, among women, the association between underweight and MACEs became more apparent as BP category increased, which may partly reflect reduced peripheral fat stores and sex-specific differences in inflammatory and vascular responses to hypertension [27, 28]. Age-stratified analyses suggested that obesity was the dominant high-risk BMI category among participants aged <40 years with stage 2/3 hypertension, whereas underweight was more prominent among those aged ≥40 years across higher BP strata. This difference may reflect obesity-related cardiometabolic burden in younger adults and, in older adults, the increasing influence of low lean mass, malnutrition, frailty, and reduced physiological reserve on BMI-based risk assessment [24, 29, 30]. These findings extend the interpretation of underweight as a cardiovascular risk marker by suggesting that its prognostic significance in the presence of hypertension may depend on sex and age, rather than representing a uniform risk pattern across all population subgroups.

The lag analysis provided additional insight into the possibility of reverse causation. After excluding early events, the overall BP-related risk gradient persisted, but the elevated HRs for underweight observed in several hypertensive categories in the main analysis were attenuated. This attenuation suggests that part of the excess risk among underweight individuals may reflect preclinical disease, frailty, low nutritional reserve, or other unmeasured health conditions present at baseline [31]. Nevertheless, underweight remained associated with increased MACE risk in higher BP categories, indicating that the association was not fully explained by early events.

These findings may inform cardiovascular risk assessment, particularly when sex and age are considered. Conventional prevention strategies for obesity with hypertension appropriately emphasize weight control and cardiometabolic risk reduction [32, 33]; however, the same approach may not be sufficient for underweight individuals with elevated BP or hypertension. This may be especially relevant for women and adults aged ≥40 years, in whom the excess risk associated with underweight and higher BP was more apparent. In these subgroups, underweight with hypertension may support additional assessment for unintentional weight loss, malnutrition, low muscle mass, frailty, and underlying chronic disease. Potential management strategies may therefore include nutritional assessment and support, resistance exercise or muscle-strengthening interventions where appropriate, and individualized BP control that considers frailty, orthostatic symptoms, and treatment tolerability. Although obesity-centered prevention remains important among men and younger adults, underweight with elevated BP or hypertension should not be regarded as low risk solely because obesity is absent. These findings suggest that underweight with hypertension may identify a clinically relevant high-risk subgroup requiring assessment beyond BP and BMI categories alone.

### Limitations

Our study has certain limitations. First, the retrospective design precludes us from establishing a cause-and-effect relationship. Despite multivariable adjustment, other conditions that both cause weight loss and increase cardiovascular risk may still confound this association. Second, the BP and BMI classifications relied on single baseline measurements, which may not fully capture intraindividual variability, potentially leading to misclassifications. Although standardized BP measurement protocols are recommended in Japanese health-check programs, adherence varies across institutions, possibly introducing measurement bias. Third, although cardiovascular outcomes were defined using validated claims-based algorithms rather than ICD-10 codes alone, outcome misclassification remains possible. Therefore, residual misclassification of MI, ischemic stroke, and ICH may have influenced the estimated associations. All-cause mortality was based on the recorded reason for loss of health insurance eligibility, and cause-specific mortality could not be evaluated. Fourth, the JMDC cohort predominantly included employed working-age individuals and their dependents enrolled in a national health insurance system, which may introduce a healthy-worker effect and limit generalizability to older adults, unemployed, or socioeconomically disadvantaged populations. Fifth, participants excluded on the basis of our criteria might have systematically differed from those analyzed, raising the potential for selection bias. Sixth, death may have acted as a competing event for individual non-fatal outcomes. Because competing-risk analyses were not computationally feasible in this very large multiply imputed dataset, the findings for individual outcomes should be interpreted as associations with event-specific hazards rather than cumulative incidence. Seventh, participants with hypertension, obesity, or underweight may have differed in their opportunities for medical evaluation, which could have introduced differential outcome ascertainment. Individuals with abnormal BP or BMI may have been more likely to undergo diagnostic testing, increasing the likelihood that cardiovascular events were captured in claims data. Although we used claims-based algorithms incorporating diagnosis, procedure, and medication codes, residual ascertainment bias cannot be excluded. Finally, data on important potential confounders, such as dietary factors, physical activity, socioeconomic status, and psychosocial stress, were unavailable in our dataset, possibly influencing the observed associations.

### Perspective of Asia

Obesity with hypertension is widely recognized as a high-risk phenotype but underweight with hypertension may also be clinically important in Asian populations. In this nationwide study of 6.26 million Japanese working-age adults, underweight participants had a relatively low crude incidence of MACEs when BP was normal, whereas MACE risk increased markedly as BP category advanced; the significant BP–BMI interaction showed that the association between BP and MACE risk was not uniform across BMI categories. This joint association also differed by sex and age, with the high-risk pattern of underweight combined with hypertension being more apparent in women and adults aged ≥40 years, while obesity remained an important risk pattern in men and younger adults. These findings suggest that Asian health-check and hypertension management programs should assess BP and BMI jointly and extend preventive assessment and appropriate intervention beyond obesity to underweight individuals with elevated BP or hypertension.

## Conclusion

In conclusion, this large nationwide cohort study showed that the relationship between BMI category and 10-year MACE risk varied significantly across BP categories, supporting the importance of evaluating BP and BMI jointly rather than separately in cardiovascular risk assessment. Underweight combined with elevated BP or hypertension was associated with increased MACE risk, particularly among women and adults aged ≥40 years. These findings suggest that underweight individuals with elevated BP or hypertension should not be regarded as low risk solely because obesity is absent, and that risk stratification should incorporate both BP and BMI categories, together with broader health status.

## Supplementary information

Supplemental Table 1

## Data Availability

The de‑identified claims and health‑check data analyzed in this study are commercially licensed from JMDC Inc. (Tokyo, Japan; https://www.jmdc.co.jp/en/index).
