# Supplementary material for: Body mass index modifies cardiovascular risk across blood pressure categories, particularly among underweight adults with hypertension: a 6.3 million‑person nationwide Japanese cohort study
Source: Hypertens Res. 2026 Aug 31;49(11):2925–35. doi: 10.1038/s41440-026-02781-8 (PMC13736646; doi:10.1038/s41440-026-02781-8)
Supplement: Supplementary file 1 [file 41440_2026_2781_MOESM1_ESM.docx]

Supplemental Table 1. Adjusted HR for individual outcomes

|  | MI | Death | Stroke |
| --- | --- | --- | --- |
| BP-BMI category | Adjusted HR (95% CI) | Adjusted HR (95% CI) | Adjusted HR (95% CI) |
| ***Normal BP*** |  |  |  |
| Underweight | 0.55 (0.41 - 0.73) | 1.56 (1.42 - 1.71) | 0.99 (0.88 - 1.12) |
| Healthy weight | 1.00 (Reference) | 1.00 (Reference) | 1.00 (Reference) |
| Overweight | 1.42 (1.25 - 1.62) | 1.11 (1.00 - 1.23) | 1.12 (1.01 - 1.25) |
| Obesity | 1.74 (1.31 - 2.31) | 1.13 (0.85 - 1.49) | 1.08 (0.81 - 1.45) |
| ***High-normal BP*** |  |  |  |
| Underweight | 1.25 (0.83 - 1.87) | 1.77 (1.47 - 2.14) | 1.24 (0.98 - 1.58) |
| Healthy weight | 1.21 (1.08 - 1.35) | 1.10 (1.02 - 1.19) | 1.24 (1.14 - 1.34) |
| Overweight | 1.65 (1.43 - 1.90) | 1.11 (0.98 - 1.26) | 1.41 (1.25 - 1.59) |
| Obesity | 1.44 (1.06 - 1.95) | 1.55 (1.21 - 1.98) | 1.61 (1.26 - 2.05) |
| ***Elevated BP*** |  |  |  |
| Underweight | 1.19 (0.82 - 1.73) | 2.05 (1.75 - 2.39) | 1.82 (1.52 - 2.18) |
| Healthy weight | 1.80 (1.65 - 1.97) | 1.22 (1.14 - 1.30) | 1.84 (1.73 - 1.96) |
| Overweight | 2.15 (1.93 - 2.39) | 1.11 (1.01 - 1.23) | 1.91 (1.75 - 2.08) |
| Obesity | 2.40 (2.03 - 2.83) | 1.30 (1.09 - 1.55) | 1.57 (1.33 - 1.85) |
| ***Stage 1 hypertension*** |  |  |  |
| Underweight | 1.58 (0.98 - 2.56) | 2.20 (1.77 - 2.74) | 2.33 (1.83 - 2.97) |
| Healthy weight | 2.13 (1.92 - 2.37) | 1.41 (1.30 - 1.53) | 2.70 (2.51 - 2.90) |
| Overweight | 2.78 (2.48 - 3.13) | 1.31 (1.17 - 1.47) | 2.95 (2.70 - 3.23) |
| Obesity | 2.72 (2.28 - 3.24) | 1.72 (1.45 - 2.05) | 2.88 (2.49 - 3.33) |
| ***Stage 2/3 hypertension*** |  |  |  |
| Underweight | 2.01 (0.96 - 4.24) | 2.97 (2.11 - 4.20) | 3.84 (2.75 - 5.37) |
| Healthy weight | 3.27 (2.85 - 3.75) | 2.21 (1.98 - 2.46) | 4.30 (3.92 - 4.72) |
| Overweight | 4.11 (3.58 - 4.73) | 1.99 (1.73 - 2.29) | 4.74 (4.26 - 5.27) |
| Obesity | 4.09 (3.36 - 4.99) | 2.86 (2.36 - 3.46) | 6.39 (5.55 - 7.35) |
|  |  |  |  |

Participants were categorized as having normal BP (untreated systolic and diastolic BPs of <120 and <80 mmHg), high normal BP (120–129 and <80 mmHg), elevated BP (130–139 and 80–89 mmHg), stage 1 hypertension (140–159 and 90–99 mmHg), or stage 2/3 hypertension (≥160 and/or ≥100 mmHg), and were further subdivided based on BMI as follows: underweight (<18.5 kg/m²), normal weight (18.5–24.9 kg/m²), overweight (25.0–29.9 kg/m²), and obesity (≥30 kg/m²).

MI, myocardial infarction; HR, hazard ratio; CI, confidence interval; BMI, body mass index; BP, blood pressure.
